# Central Retinal Vein Occlusion Following the First Dose of COVID Vaccine

**DOI:** 10.7759/cureus.25842

**Published:** 2022-06-11

**Authors:** Punita K Sodhi, Alka Yadav, Bhumika Sharma, Anu Sharma, Prashant Kumar

**Affiliations:** 1 Ophthalmology, Guru Nanak Eye Centre and Maulana Azad Medical College, New Delhi, IND; 2 Ophthalmology, Dr. Rajendra Prasad Centre for Ophthalmic Sciences, All India Institute of Medical Sciences, New Delhi, IND

**Keywords:** retina and vaccine, post vaccination thromboembolism, vaccine adverse effects, covid vaccine, central retinal vein occlusion

## Abstract

The reports of vascular adverse events in the eye following COVID-19 vaccination are infrequent. We report the case of a healthy male who developed central retinal vein occlusion in his left eye three days following administration of the first dose of Covishield vaccine. As the underlying systemic and ocular risk factors were absent and laboratory investigations were normal, vein occlusion appeared to probably result from the vaccine. The patient developed retinal hemorrhages and non-perfusion ischemic areas all over the fundus. The macular edema was reduced with intravitreal triamcinolone acetonide, but the visual gain was not much, which appears to be due to the time lag in his initial presentation to the Ophthalmology Department. A close watch should be kept for ophthalmic adverse events to have an early intervention.

## Introduction

India is running the largest vaccination campaign against coronavirus disease 2019 (COVID-19), mainly with the Oxford-AstraZeneca COVID-19 vaccine (AZD1222; known locally as Covishield) and Covaxin (BBV152) vaccine. The COVID-19 vaccines can cause neurological, cardiovascular, otorhinolaryngological, and vascular adverse events attributed to thromboembolic, inflammatory, immunological, or anaphylactic mechanisms precipitated by the vaccine [1,2].

We report a case of a healthy young man who developed central retinal vein occlusion (CRVO) in the left eye (LE) within three days of receiving his first dose of the Covishield COVID-19 vaccine. CRVO is associated with risk factors such as raised intraocular pressure (IOP), hypertension, diabetes, cardiovascular disease, hypercoagulable state, inflammation, deranged lipid profile, and elevated serum homocysteine levels. These factors lead to a compression of the central retinal vein or obstruction in its blood flow from a thrombus or an embolus, causing ischemia, edema, and, finally, neovascularization [3]. Though an underlying or undiagnosed illness independent of vaccination may also be a causative factor, the emergence of complications within a short period from vaccination suggests the latter [4]. A search of the PubMed Index in different languages shows that few cases of CRVO after COVID-19 vaccination have been reported [4-7].

## Case presentation

A 43-year-old healthy man developed sudden painless diminution of his vision in the LE three days after receiving the first dose of the Covishield COVID-19 vaccine (Batch No. 4121MC028; Expiry October 31, 2021). He had no history of smoking, ocular surgery, systemic illness, trauma, excessive physical exercise, or dehydration. He was diagnosed with CRVO in the LE at a separate clinic. Two months later, he presented to our ophthalmology outpatient department and tested negative on reverse transcriptase-polymerase chain reaction for COVID-19. His blood pressure was 130/90 mmHg. His laboratory investigation results were within reference ranges except for glycated hemoglobin, which was slightly raised (Table 1). His electrocardiogram and echocardiography study revealed no ischemia or any other significant findings.

**Table 1:** Laboratory investigations of the patient with reference ranges P, polymorphonuclear leucocytes; L, lymphocytes; M, monocytes; E, eosinophils, B: basophils

Analyte	Patient’s Value	Reference Range
Hemoglobin	14.3 gram/deciliters	13-17 gram/deciliters
Blood sugar fasting	67 milligrams/deciliters (slightly low)	70-99 milligrams/deciliters
Blood sugar post prandial	95 milligrams/deciliters (slightly low)	120-180 milligrams/deciliters
Acetylated hemoglobin	6.7% (slightly raised)	<6.5%
Serum glutamic oxaloacetic transaminase	22 units/liter	<35 units/liter
Serum glutamic pyruvic transaminase	32 units/liter	<45 units/liter
Blood urea	34 milligrams/deciliters	15-40 milligrams/deciliters
Serum creatinine	1.0 milligrams/deciliters	0.6-1.1 milligrams/deciliters
Total leucocyte count	6.98 x 10^3^/microliters	4-10 x 10^3^/microliters
Differential leucocyte count	P63 L30 M4 E3 B0	
Platelet count	187 x 10^3^/microliters	150-400 x 10^3^/microliters
Bleeding time	3 minutes	2-5 minutes
Clotting time	4 minutes	3-6 minutes
Prothrombin time	11.5 seconds	11-13.5 seconds
Activated partial thromboplastin time	25 seconds	21-35 seconds
Blood lipid profile		
High-density lipoproteins	39 milligrams/deciliters	40-60 milligrams/deciliters
Triglycerides	140 milligrams/deciliters	<150 milligrams/deciliters
Serum homocysteine	14 micromoles/Liters	5-15 micromoles/Liters
C-reactive protein	2.1 milligrams/Liters	<5 milligrams/Liters
D-dimer	200 nanograms/milliliters	<500 nanograms/milliliters

**Figure 1:**
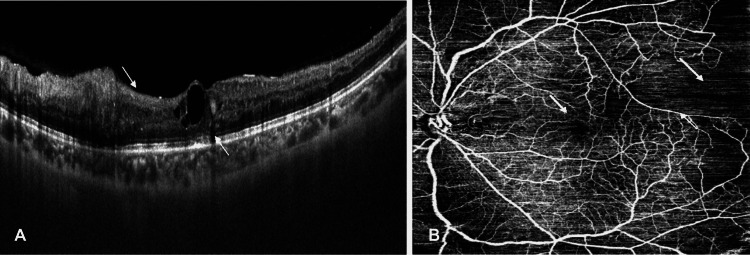
Optical coherence tomography scan of the left eye (arrows show disorganized retinal inner layers and disruption of inner segment-outer segment junction). (B) Optical coherence tomography angiography scan of the left eye (arrows show enlarged foveal avascular zone and capillary non-perfusion).

The ocular examination showed that he had best-corrected visual acuity (BCVA) of logarithm of the minimum angle of resolution (LogMAR) +0.2 (20/32, 6/9) in the right eye (RE) and LogMAR +1.5 (20/630, 6/190) in the LE. The pupils in the LE reacted sluggishly to the light reflex test. His IOP was 16 mmHg in both eyes. The anterior chamber had normal depth and open angles. There was no neovascularization of the iris or angle. The fundus examination showed optic disc edema, dilated tortuous veins, dot-blot and flame-shaped hemorrhages across the fundus, and blunted foveal reflex in the LE. Findings in the RE were unremarkable. The optical coherence tomography (OCT) in the LE showed disorganization of the inner retinal layers, disruption of the inner segment-outer segment junction, and distorted foveal contour with large cystic spaces. The central foveal thickness (CFT) measured 854 microns. The foveal contour was normal in the RE, and the CFT measured 242 microns. The OCT angiography showed an enlarged distorted foveal avascular zone (FAZ) and capillary non-perfusion in a 12-mm x 12-mm scan in the LE (Figure 1) and normal-sized circular FAZ in the RE.

**Figure 2:**
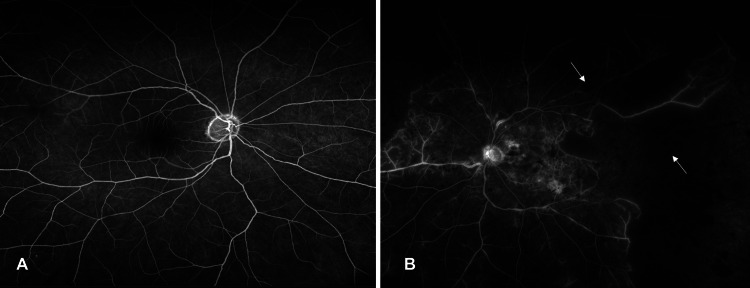
(A) Fundus fluorescein angiography of the right eye. (B) Fundus fluorescein angiography of the left eye (arrows show blocked fluorescence and capillary non-perfusion).

The fundus fluorescein angiography (FFA) revealed that the LE had delayed venous filling, blocked fluorescence from retinal hemorrhages, and capillary non-perfusion areas extending through 360 degrees in the peripheral fundus but no neovascularization of disc or elsewhere (Figure 2). The FFA of the RE was normal. The flash electroretinogram showed the scotopic and photopic amplitudes of the b-wave at 230 and 110 microvolts in RE and 80 and 50 microvolts in LE, respectively. 

We established a diagnosis of ischemic CRVO and cystoid macular edema (CME) in the LE [3]. The RE was unremarkable. He was given an intravitreal injection of triamcinolone acetonide (4 mg/0.1 ml) in the LE for CME. One month later, the LE had BCVA of LogMAR +1.2 (20/320, 6/95), and the CFT measured 652 micrometers. The patient presented again six months after the initial episode. He had BCVA of LogMAR +1.0 (20/200, 6/60). His fundus examination showed that hemorrhages and exudates had resolved. The CFT had reduced to 320 micrometers. The FFA showed capillary non-perfusion areas extending through 360 degrees in the peripheral fundus and one neovascularization elsewhere (NVE) in the superonasal quadrant. A targeted laser was performed for ischemic areas and the NVE.

## Discussion

The vascular adverse events in the eye following COVID-19 vaccination have been observed infrequently [2]. Sonawane et al. reported two cases of ischemic CRVO that presented three to four days after the second dose of the COVID-19 vaccine (Covishield, AZD1222, ChAdOX 1) [5]. The first case was a 50-year-old man with uncontrolled diabetes and deranged renal functions whose RE had ischemic CRVO, and CME and LE showed mild non-proliferative diabetic retinopathy changes. Their second case was a 43-year-old woman with no systemic disease who presented with an elevated erythrocyte sedimentation rate of 49 mm/hour, C-reactive protein of 14.6 mg/L (reference range: <5 mg/L), a rheumatoid factor of 11 IU/mL (reference range: <8 IU/mL) and d-dimer of 6077.4 ng/mL (reference range, <500 ng/mL). The involved RE did not show CME at presentation [5]. Bialasiewicz et al. described a case of an otherwise healthy 50-year-old man with ischemic CRVO presenting with sudden, painful diminution of vision immediately after the second dose of the COVID-19 vaccine (BioNTech/Pfizer lot number EP6017) [6]. Endo et al. a case of reported a 52-year-old healthy man who developed non-ischemic CRVO 15 days after receiving the first dose of the vaccine (Pfizer-BioNTech) [7]. Ikegami et al. reported a case of combined central retinal artery and vein occlusion in the RE two days after the second dose of the vaccine (mRNA-1273) in a 54-year-old hypothyroid woman [4]. In both cases, various laboratory tests on coagulation, antibody, lipid, and glucose profiles were within reference ranges [4,7].

AstraZeneca's COVID-19 vaccine has been associated with an increased risk of blood clots in combination with low levels of blood platelets (a percentage of risk being 0.0007%) [8]. Even after the first dose of vaccination with ChAdOX 1 (Covishield), Simpson et al. reported a high incidence of adverse effects (1.13 cases per 100,000 vaccinations) such as immune thrombocytopenic purpura and arterial thromboembolic and hemorrhagic events [9]. While CRVO in patients over 65 years of age is associated with systemic vascular conditions such as diabetes or hypertension, younger patients usually have an underlying hypercoagulable or inflammatory etiology [3]. Our patient was a healthy young man who developed CRVO following the first dose of the Covishield vaccine. The appearance of CRVO shortly after vaccination suggests an association between the two events.

## Conclusions

While rare, ocular events can occur following the administration of the COVID-19 vaccine. This case described an otherwise healthy man who developed sight-threatening complications following the first dose of the Covishield vaccine. Vigilance for ophthalmic adverse events following vaccine administration is required for early intervention.
